# Supplementary material for: Deep-Fed: A comprehensive solution for precise bone fracture identification in athletes
Source: PLoS One. 2026 Mar 12;21(3):e0343980. doi: 10.1371/journal.pone.0343980 (PMC12981491; doi:10.1371/journal.pone.0343980)
Supplement: Attachment — Submitted filename: PONE-D-25-34607 Editor Comments.docx [file pone.0343980.s001.docx]

Based on a thorough review of the manuscript "**Deep-Fed: A Comprehensive Solution for Precise Bone Fracture Identification in Athletes**" (Manuscript Number: PONE-D-25-34607), the following six major review comments are suggested for revision to enhance the quality, clarity, and scientific rigor of the submission:

1. **Abstract Completeness**: The abstract is significantly truncated, limiting the ability to assess the full scope of the study, including methodology, results, and implications. The authors should provide a complete abstract (within the 300-word limit per PLOS ONE guidelines) that concisely summarizes the background, objectives, methods (e.g., federated deep learning with ConvNet and FractureNet), key results (e.g., accuracy rates across all datasets), and the significance of Deep-Fed for sports medicine.
2. **Data Availability Statement**: The manuscript mentions three datasets (Deep-I, Deep-II, Deep-III) but lacks a clear Data Availability Statement as required by PLOS ONE. The authors must confirm whether the data underlying the findings are fully available without restriction and provide specific details (e.g., repository links, dataset names, or a statement if data are within the manuscript/supporting files). If data sharing is restricted, a detailed explanation and contact information for access approval must be included.
3. **Mathematical and Algorithmic Clarity**: The manuscript contains incomplete or erroneous mathematical expressions and algorithms (e.g., truncated equations on Pages 19, 20, 24, and incomplete Algorithm 4 on Page 27). The authors should revise and complete all mathematical formulations (e.g., normalization, convolution, pooling operations) and provide full, pseudocode for Algorithms 1-4, ensuring they are executable and aligned with the described methodology.
4. **Consistency in Results**: The reported accuracy rates vary across the manuscript (e.g., 96.23%, 97.11%, 96.73% on Page 10 vs. 95.6%, 97.34%, 96.12% on Page 30 vs. 97.23%, 98.11%, 98.23% on Page 33). The authors need to reconcile these discrepancies, provide a single, consistent set of results, and justify any variations through additional analysis or experimental conditions.
5. **Literature Comparison and Table Completion**: Table 1 on Page 16 is incomplete, with only one reference ([25]) detailed. The authors should expand this table to include all cited works (e.g., [23], [24]) with their core methods, accuracy, and limitations, ensuring a comprehensive comparison with Deep-Fed. Additionally, the literature review should be updated to reflect how Deep-Fed addresses the identified gaps more effectively.
6. **Ethical and Funding Details**: The manuscript states "N/A" for the ethics statement and provides a partial funding acknowledgment (Northern Border University, Arar, KSA) without full details. The authors must either provide a detailed ethics statement (if applicable, e.g., IRB approval for dataset use) or justify why it is not required. For funding, a complete financial disclosure statement (including author initials, grant numbers, funder URLs, and sponsor roles) must be included as per PLOS ONE requirements.
