# Supplementary material for: Deep-Fed: A comprehensive solution for precise bone fracture identification in athletes
Source: PLoS One. 2026 Mar 12;21(3):e0343980. doi: 10.1371/journal.pone.0343980 (PMC12981491; doi:10.1371/journal.pone.0343980)
Supplement: Attachment — Submitted filename: Response to Reviewers.docx [file pone.0343980.s003.docx]

**Response to Editor’s Comments**

**Title: Deep-Fed: A Comprehensive Solution for Precise Bone Fracture Identification in Athletes**

We are very thankful to the editor for his decision and reviewers for their valuable suggestions. We have revised the whole manuscript, incorporated all the comments, and highlighted the changes in the revised draft. We hope that the revised manuscript is now according to the required standard. The response to the reviews is summarized as below:

| **S. No.** | **Reviewers Comments** | **Author Reply:** |
| --- | --- | --- |
| **Editor Comments** | | |
| 1 | Based on a thorough review of the manuscript "Deep-Fed: A Comprehensive Solution for Precise Bone Fracture Identification in Athletes" (Manuscript Number: PONE-D-25-34607), the following major review comments are suggested for revision to enhance the quality, clarity, and scientific rigor of the submission:  1. Abstract Completeness: The abstract is significantly truncated, limiting the ability to assess the full scope of the study, including methodology, results, and implications. The authors should provide a complete abstract (within the 300-word limit per PLOS ONE guidelines) that concisely summarizes the background, objectives, methods (e.g., federated deep learning with ConvNet and FractureNet), key results (e.g., accuracy rates across all datasets), and the significance of Deep-Fed for sports medicine. | .  The abstract has been revised to provide a comprehensive summary of the study, including the background, objectives, methodology (federated deep learning with ConvNet and FractureNet), key results (accuracy rates across all datasets), and the significance of Deep-Fed for sports medicine, in accordance with the PLOS ONE guidelines. |
| 2 | Data Availability Statement: The manuscript mentions three datasets (Deep-I, Deep-II, Deep-III) but lacks a clear Data Availability Statement as required by PLOS ONE. The authors must confirm whether the data underlying the findings are fully available without restriction and provide specific details (e.g., repository links, dataset names, or a statement if data are within the manuscript/supporting files). If data sharing is restricted, a detailed explanation and contact information for access approval must be included. | The datasets are publicly available, and the details have been incorporated into the manuscript. |
| 3 | Mathematical and Algorithmic Clarity: The manuscript contains incomplete or erroneous mathematical expressions and algorithms (e.g., truncated equations on Pages 19, 20, 24, and incomplete Algorithm 4 on Page 27). The authors should revise and complete all mathematical formulations (e.g., normalization, convolution, pooling operations) and provide full, pseudocode for Algorithms 1-4, ensuring they are executable and aligned with the described methodology. | All incomplete mathematical expressions have been revised and corrected, and Algorithms 1–4 have been completed with full pseudocode to ensure clarity and alignment with the methodology. |
| 4 | Consistency in Results: The reported accuracy rates vary across the manuscript (e.g., 96.23%, 97.11%, 96.73% on Page 10 vs. 95.6%, 97.34%, 96.12% on Page 30 vs. 97.23%, 98.11%, 98.23% on Page 33). The authors need to reconcile these discrepancies, provide a single, consistent set of results, and justify any variations through additional analysis or experimental conditions. | The accuracy have now been carefully verified and updated in the manuscript to ensure consistency across all sections. Any discrepancies were resolved, and a single, consistent set of results is now presented. |
| 5 | 5. Literature Comparison and Table Completion: Table 1 on Page 16 is incomplete, with only one reference ([25]) detailed. The authors should expand this table to include all cited works (e.g., [23], [24]) with their core methods, accuracy, and limitations, ensuring a comprehensive comparison with Deep-Fed. Additionally, the literature review should be updated to reflect how Deep-Fed addresses the identified gaps more effectively. | Table 1 has been updated in the manuscript to include all relevant references ([23], [24], [25]) along with their core methods, reported accuracy, and limitations. Furthermore, the literature review has been revised to provide a more comprehensive comparison, clearly highlighting how Deep-Fed addresses the identified research gaps more effectively. |
| 6 | Ethical and Funding Details: The manuscript states "N/A" for the ethics statement and provides a partial funding acknowledgment (Northern Border University, Arar, KSA) without full details. The authors must either provide a detailed ethics statement (if applicable, e.g., IRB approval for dataset use) or justify why it is not required. For funding, a complete financial disclosure statement (including author initials, grant numbers, funder URLs, and sponsor roles) must be included as per PLOS ONE requirements. | The funding acknowledgment has been updated to include complete details, including the grant number. Moreover, the ethics statement clarifies that IRB approval was not required since the datasets used are publicly available |
| Reviewer 1 | | |
| 1 | Title: Deep-Fed: A Comprehensive Solution for Precise Bone Fracture Identification in Athletes The paper seems technically good. It has proposed an innovative prediction of bone fractures by using federated deep learning framework. The framework integrates deep learning techniques within a privacy-preserving structure. It involves standardized data preprocessing, data augmentation to improve robustness, and feature extraction using a convolutional neural network. The diagnosis is performed by the FractureNet model, while federated learning ensures training across multiple sources without sharing raw data, maintaining patient privacy  Suggestions for improvements:  Introduction: In the Introduction section, please incorporate a few recent references into the opening paragraphs. Additionally, the citation sequence is currently incorrect; references should begin from [1], not [5]. Ensure that all factual claims and prior findings—such as "X-rays, though effective in detecting obvious breaks, may miss 61 subtle or hairline fractures, especially in complex regions of anatomy such as the wrists or feet.”—are properly cited.  Moreover, revise the transitions between paragraphs to improve the logical flow and narrative consistency within the Introduction.  In the Contribution section, the proposed model is referred to as Dual-Deep, whereas the title mentions Deep-Fed. Please use a consistent model name throughout the manuscript, aligned with the one used in the title.  Lastly, ensure the entire paper is thoroughly proofread for grammatical consistency and clarity. | Thank you for your valuable feedback and appreciation of our work.  In the revised manuscript, we have incorporated recent references into the opening paragraphs of the Introduction and ensured that the citation sequence now begins correctly from [1]. All factual claims and prior findings, including the statement regarding X-rays missing subtle or hairline fractures, have been properly cited. Additionally, we have revised the transitions between paragraphs to improve logical flow and narrative consistency within the Introduction.  Furthermore, the inconsistency in the model name has been addressed. The manuscript has been updated to consistently use Deep-Fed, aligning with the title.  The entire manuscript has been thoroughly proofread and revised to ensure grammatical consistency, clarity, and improved readability. |
| 2 | Literature Review: Some of the papers discussed in the Literature Review are missing from the comparison table. For instance, please include “Cao et al. [24] – SAFracNet” and “Cheng et al. [22]” in the comparison table for completeness. Additionally, revise and clarify certain sentences toward the end of this section.  For example, consider rephrasing the following for improved clarity and coherence: “The scope therefore covers all aspects—from the collection of comprehensive data to input standardization via preprocessing. Furthermore, data augmentation is applied to improve model robustness, followed by feature extraction using a ConvNet architecture, and final diagnosis by the FractureNet model. Each of these steps ensures data privacy and security through federated learning.” | In the revised manuscript, we have updated the comparison table to include the missing works, specifically Cao et al. [24] – SAFracNet and Cheng et al. [22], ensuring completeness and consistency across the Literature Review.  Additionally, we have revised and clarified the concluding sentences of this section to improve clarity and coherence. |
| 3 | Methodology: The term Dual-Deep is again used here instead of the correct model’s name Deep-Fed. Please ensure consistent terminology throughout the paper. At the beginning of the Methodology section, provide a brief summary or overview of the proposed framework as illustrated in Figure 1. Please add further details about Federated Learning for more clarity on the setup and configuration. Also, ensure that all text in figures is readable and the images are of high quality. Currently, some figures are blurry and difficult to read. | We have ensured consistent use of the correct model name Deep-Fed throughout the paper.  At the beginning of the Methodology section, a brief summary and overview of the proposed framework, as illustrated in Figure 1, has been added to provide better context.  Further details about the federated learning setup and configuration have also been incorporated for greater clarity.  Additionally, all figures have been updated with improved resolution and formatting to ensure that the text is clear and fully readable. |
| 4 | Results: Some of the baseline studies used for comparison in this section are not listed in the comparison table of the Literature Review. Please update the table accordingly. Consider replacing or supplementing the current table with a line graph showing log loss for better visual interpretation. In addition, including a ROC curve would enhance the presentation and interpretation of your model’s performance. | The comparison table in the Literature Review has been updated to include all the baseline studies discussed in this section, ensuring completeness and consistency. Additionally, a line graph illustrating log loss has been incorporated to supplement the table, providing a clearer visual interpretation of the comparative results. |
| 5 | References:  Please include a few recent references from the year 2025 to reflect the current state of research. | In the revised manuscript, a few recent references from 2025 have been incorporated to better reflect the current state of research and ensure the literature review remains up to date. |
| Reviewer 2 | | |
| 1 | Reviewer #2: The manuscript introduces Deep-Fed, a federated deep learning framework for bone fracture detection, targeting athlete-specific applications. The concept of integrating federated learning with deep CNN-based feature extraction is relevant and addresses current challenges in medical imaging privacy and robustness. The manuscript is structured well, but there are several concerns regarding technical clarity, experimental transparency, and reproducibility that need to be addressed. Major Comments  o The paper does not include code availability, architectural specifics, or training logs, making reproduction difficult. | Thank you for your valuable feedback and appreciation of our work.  We have added further details regarding the architectural specifications of Deep-Fed, the training configurations and logs. These additions improve transparency and enable future researchers to replicate our results more effectively. |
| 2 | The manuscript mentions three datasets (Deep-I, II, III) but lacks clarity on: - Whether these datasets were used in federated (distributed) settings or centrally. - How the data was partitioned (e.g., balanced, unbalanced). | The three datasets (Deep-I, Deep-II, and Deep-III) were used in a federated learning setting, with data distributed across simulated clients. Partitioning followed a non-IID strategy with stratified sampling to balance fracture-positive and negative cases, ensuring realistic heterogeneity in evaluation. |
| 3 | The manuscript claims privacy preservation via federated learning but does not provide empirical privacy evaluations (e.g., differential privacy noise impact, data leakage checks). | Thank you for the insightful comment. While this study primarily focused on evaluating the diagnostic performance of Deep-Fed, we acknowledge that empirical privacy evaluations (e.g., impact of differential privacy noise or leakage analysis) were not conducted. In future work, we plan to extend the framework with quantitative privacy assessments to rigorously evaluate trade-offs between privacy preservation and model performance. |
| 4 | Baselines are compared in terms of classical metrics, but recent federated learning frameworks for medical imaging are not cited or compared (e.g., FedAvg, FedProx). | We have now included recent federated learning frameworks for medical imaging, such as FedAvg and AdaFedProx, and provided their comparative results alongside our proposed model to ensure a comprehensive evaluation |
| 5 | Minor Comments  o Terms like FL, ConvNet, CNN, FedAvg appear before definition in some places. o Phrases like "extremely accurate", "overwhelmingly outperforming" are too subjective. o Figures (e.g., Figure 4–6) are not thoroughly discussed. Some could be merged or clarified. o Table 2 mistakenly labels two columns as "Baseline 2". o Frequent grammatical issues such as "the author have" instead of "authors have", inconsistent article usage. | All abbreviations (e.g., FL, ConvNet, CNN, FedAvg) are now defined before first use, and subjective phrases have been replaced with objective descriptions. Figures have been revised with clearer discussion, merging where appropriate, and Table 2 has been corrected. In addition, the manuscript has undergone thorough grammatical revision to address errors and ensure consistency. |
