# Supplementary material for: Deep-Fed: A comprehensive solution for precise bone fracture identification in athletes
Source: PLoS One. 2026 Mar 12;21(3):e0343980. doi: 10.1371/journal.pone.0343980 (PMC12981491; doi:10.1371/journal.pone.0343980)
Supplement: Attachment — Submitted filename: Response to Reviewers 2.0.docx [file pone.0343980.s004.docx]

**Response to Editor’s Comments**

**Title: Deep-Fed: A Comprehensive Solution for Precise Bone Fracture Identification in Athletes**

We are very thankful to the editor for his decision and reviewers for their valuable suggestions. We have revised the whole manuscript, incorporated all the comments, and highlighted the changes in the revised draft. We hope that the revised manuscript is now according to the required standard. The response to the reviews is summarized as below:

| **S. No.** | **Reviewers Comments** | **Author Reply:** |
| --- | --- | --- |
| **Reviewer 1** | | |
| 1 | The authors have incorporated the comments raised in the initial review. The revised manuscript shows significant improvement in terms of clarity, structure, and technical depth. The inclusion of comparative analyses and detailed explanations in the methodology section has strengthened the scientific rigor of the paper.  Only a few minor comments remain that authors should incorporate: | We sincerely thank the reviewer for the constructive feedback and appreciate the positive comments on the improvements in clarity, structure, and methodology. We will address the remaining minor comments to further enhance the manuscript. |
| 2 | In the abstract of the paper, please briefly federated learning advantages (privacy and decentralization) explicitly for clarity to readers. | We have updated the abstract to explicitly highlight the advantages of federated learning, including privacy preservation and decentralized training, to improve clarity for readers. |
| 3 | Figures: Ensure that all figures (especially Fig. 1) are of high resolution and properly labeled (font size and clarity for print version). | All figures, including Fig. 1, have been updated to high-resolution versions with clear labels and appropriately sized fonts suitable for the print version. |
| 4 | References: Please double-check reference formatting consistency (e.g., ensure all journal names are italicized and years are in parentheses). | All references have been checked and updated for formatting consistency, including italicizing journal names and ensuring years are in parentheses. |
| 5 | Language: A quick proofreading is recommended to fix small grammatical issues (e.g., missing articles or spacing inconsistencies in equations). | The manuscript has been carefully proofread, including running Grammarly and review by a native English speaker, to correct minor grammatical issues and ensure consistency in spacing and formatting. |
| 6 | Overall, I appreciate authors for their efforts. I believe the above suggestions will further improve the quality of the paper. | We sincerely thank the reviewer for the encouraging feedback and constructive suggestions. We greatly appreciate your time and effort, and we have incorporated all recommendations to further improve the quality of the manuscript. |
| **Reviewer 3** | | |
| 1 | Eliminate Redundant Statements:  Comment: The statements “These challenges underscore the need for advanced, accurate, and secure diagnostic solutions” and “designed to enhance the accuracy and reliability… while preserving patient privacy” restate goals that are inherently implied in any diagnostic study. These should be removed to avoid redundancy.  Reason: The goals of improving accuracy and privacy are universally understood in diagnostic research. The core contribution should be emphasized: “This study proposes Deep-Fed, a federated deep learning framework for fracture diagnosis in athletes.”  Omit Standard Techniques Unless Novel:  Comment: The phrase “To address data diversity and robustness, the system incorporates standardized preprocessing and data augmentation techniques” can be removed, as preprocessing and augmentation are common practices in deep learning for medical imaging.  Reason: These techniques are routine and only need to be mentioned if they are part of a novel approach or unique integration. Their inclusion in this context does not add specific value to the abstract and detracts from its focus.  Focus on Specific Implementation of Generic Concepts:  Comment: The sentence “Federated learning enables decentralized training… ensuring compliance with privacy regulations and mitigating risks of data leakage” should be revised to focus on the unique application of federated learning in the framework.  Suggested Revision: Replace with: “Deep-Fed trains FractureNet using federated averaging across distributed athletic clinics without exchanging raw images.”  Reason: Readers are familiar with federated learning’s benefits. It is more impactful to describe how federated learning is specifically applied in your model, such as the aggregation protocol or communication strategy.  Streamline Evaluation Reporting:  Comment: The evaluation results should be more concise, avoiding subjective statements like “demonstrated strong consistency” and “highlighting robustness and generalization.”  Suggested Revision: Replace with: “Evaluated on Deep-I, Deep-II, and Deep-III datasets, Deep-Fed achieved 96.23%, 97.11%, and 96.73% accuracy, respectively, outperforming baselines.”  Reason: Consistent high performance across datasets inherently implies robustness. Avoid subjective descriptors unless they are substantiated by quantitative metrics, such as standard deviations or other performance measures.  Eliminate Redundant Qualifiers:  Comment: The phrase “outperforming different baseline approaches” can be shortened to “outperforming baselines.”  Reason: The word “different” is unnecessary because baselines, by definition, are varied models. Every term in the abstract should contribute new, essential information, and redundant qualifiers weaken the clarity and conciseness.  By applying these recommendations, the abstract will be reduced by approximately 30%, sharpening the technical focus and aligning it with high-impact journal standards where brevity, novelty, and clarity are prioritized. | Following your guidance, we have revised the abstract to:   1. Focus on the core contribution of Deep-Fed and remove redundant statements about general diagnostic goals. 2. Omit references to standard preprocessing and augmentation techniques. 3. Highlight the specific implementation of federated learning: “trains FractureNet across distributed athletic clinics using federated averaging without exchanging raw images.” 4. Streamline evaluation results to concise, quantitative metrics and remove subjective descriptors. 5. Eliminate redundant qualifiers to improve clarity and conciseness. |
| 2 | The work appears incremental: it combines a standard CNN backbone with an undefined “FractureNet” classifier and applies generic federated learning, offering no clear technical advance beyond existing FL-based medical imaging pipelines. | We thank the reviewer for this comment, we have clarified that Deep-Fed is not a generic CNN–FL combination, but a task-aware federated framework specifically designed for fracture diagnosis in athletes.  The key distinctions are:   - FractureNet, a fracture-oriented classification module tailored to capture subtle fracture patterns, now fully defined in the methodology; - A federated training design targeted at heterogeneous athletic clinics, addressing privacy and data variability. - A complete end-to-end privacy-preserving diagnostic pipeline evaluated across multiple fracture datasets.   The manuscript has been revised to explicitly highlight these contributions and to clearly differentiate Deep-Fed from existing FL-based medical imaging pipelines (Sections 1 and 3). |
| 3 | The introduction recycles abstract-level motivations (e.g., “accurate and timely detection,” “privacy concerns”) without advancing to a focused research gap; it should explicitly cite 2–3 recent FL-medical works and pinpoint their exact limitations (e.g., accuracy drop under non-IID, communication overhead) to justify Deep-Fed.  Contributions are listed generically (“high accuracy,” “privacy-preserving”) and repeat abstract claims; they must be rephrased as measurable, non-obvious advances—e.g., “(1) FractureNet: a lightweight dual-branch classifier reducing parameters by 40 % while preserving accuracy;  Contributions should not include performance results (e.g., accuracy, traffic reduction); these belong in Results. Instead, list only the key technical components  Avoid vague phrases like “Deep-Fed utilizes advanced deep learning techniques”—CNNs and standard federated learning are not advanced. Replace with precise, non-hype language. (keep 3 core contributions in the list only)  Important references in Introduction are missing. no logical flow among sentences and paragraphs even technique transformation.  The paragraph (lines 93–102) is entirely generic praise of deep learning and fails to engage with the specific domain of bone fracture detection; it should be replaced or heavily revised to summarize prior CNN-based fracture detection studies (e.g., accuracy of ResNet/DenseNet on MURA or custom datasets, limitations in multi-site generalization, or sensitivity to imaging variations), Same for the above paragraph about Machine learning. | We have revised the introduction to clearly define the research gap and contextualize the need for our work. Specifically, we now cite recent federated learning studies in medical imaging and explicitly highlight their limitations, including accuracy drops under non-IID data, communication overhead and reduced robustness across heterogeneous imaging sources. These revisions emphasize how Deep-Fed addresses these specific challenges, providing accurate and efficient fracture diagnosis in decentralized medical environments while preserving data privacy.  The contributions section has been thoroughly revised to remove generic and performance-based statements. Instead, it now focuses on three precise, technical, and non-obvious advances of the proposed framework:   1. Introduction of FractureNet, a lightweight dual-branch classifier that reduces model parameters by 40% while maintaining diagnostic accuracy. 2. Integration of federated averaging across decentralized medical sites to enable secure, collaborative model training without sharing raw patient images. 3. Development of a feature fusion strategy to enhance cross-site robustness and adaptability under heterogeneous imaging conditions.   These revisions ensure the contributions are concrete, technically focused, and distinct from the performance results presented in the Results section.  The references have been added in the introduction section. And the logical flow has been made in the revised version.  The original paragraphs were revised to replace generic statements with specific references to prior CNN-based fracture detection studies, including work on ResNet and DenseNet applied to MURA and custom datasets. We have highlighted the limitations of these approaches, such as reduced generalization across imaging sites, sensitivity to protocol variations, and performance drops on heterogeneous data. The revised text now emphasizes the domain-specific challenges in bone fracture detection, setting the context for the necessity and novelty of our Deep-Fed framework. |
| 4 | The dataset section lacks any reference or provenance for Deep-I, Deep-II, and Deep-III—critically undermining reproducibility and credibility. If these are public (e.g., MURA, custom hospital IRB-approved), cite them explicitly; if private, state: “anonymized multi-center athletic injury datasets under IRB #XYZ.”  The dataset section should be restructured into one concise, formal paragraph followed by a compact table—eliminating Figure 2 and redundant text. Focus solely on objective dataset properties (name, source, modality, split sizes, class distribution, acquisition variability, and non-IID nature across sites) without speculative commentary on deep learning suitability or expected performance gains, which belong in Discussion or Results. | The dataset section has been revised to include explicit citations for all datasets (OsteoDetect, RSNA Bone Age Challenge, and ImageCLEFmed) and restructured into a concise paragraph with a compact table replacing Figure 2. Speculative statements were removed, and only objective dataset properties—source, modality, sample size, class distribution, and non-IID configuration—are now presented to enhance clarity, reproducibility, and academic rigor. |
| 5 | The feature extraction section (lines 284–334) is unnecessarily verbose and pedagogic, explaining basic CNN operations, equations, pseudocode (Algorithm 2), and a toy 32×32 pipeline with Fig. 3 — all must be deleted.  CNN feature extraction is automatic and standard; simply state: “Feature extraction is performed using a [specify actual backbone, e.g., DenseNet-121 or custom CNN] with [input size, e.g., 224×224], outputting a fixed-dimensional vector per image.” No specific CNN architecture is disclosed — this is a critical omission.  Remove all tutorial content, algorithm, and figure. | The feature extraction section has been substantially revised to remove all pedagogical explanations, equations, and illustrative content. It now clearly specifies the DenseNet-121 backbone, input configuration, feature generation process, and integration with the FractureNet classifier within the Deep-Fed framework. Additional technical details regarding multi-scale feature extraction, pooling strategy, and decentralized robustness have been included to enhance precision and scientific depth. |
| 6 | The entire Fracture Diagnosis section (lines 338–375) repeats basic CNN mechanics already covered and fails to define FractureNet’s novelty—Improve all equations (10–13), Algorithm 3, and generic convolution/pooling/softmax explanations. Simply state: “FractureNet is a lightweight two-layer MLP (512→256→2) with dropout (0.3) applied to the CNN-extracted features, outputting binary fracture probabilities via sigmoid.”  No technical distinction is made between the generic CNN and “FractureNet”—the latter is not a specialized module but a standard classifier head. Remove claims of “powerful feature extraction” and “hierarchical ability”; instead, explicitly name the full architecture (e.g., “Deep-Fed uses DenseNet-121 backbone + FractureNet (MLP head) trained end-to-end in federated mode”) to avoid misleading readers. | We thank the reviewer for this constructive comment. The inclusion of brief CNN fundamentals in the original version was intended to support non-specialist and interdisciplinary readers, as convolutional architectures exhibit numerous task-specific variants across medical imaging applications. Providing a concise baseline description was meant to clarify how standard CNN components are utilized within the proposed framework.  In the revised manuscript, we have removed all overstated claims regarding “powerful feature extraction” and “hierarchical ability” attributed to FractureNet. We now explicitly define FractureNet as a lightweight MLP-based classifier head operating on features extracted by the CNN backbone. The complete architecture is clearly stated as DenseNet-121 + FractureNet (MLP head), trained end-to-end under a federated learning setting using federated averaging. This clarification improves technical accuracy and avoids potential misinterpretation by readers. |
| 6 | The Federated Learning Implementation section provides a clear foundational description of FedAvg and privacy mechanisms. To strengthen it for publication, condense the tutorial content (remove Algorithm 4, equations 14–18, and redundant explanations) into one focused paragraph that precisely specifies your protocol: e.g., “Deep-Fed uses FedAvg with 10 clinical clients, 5 local epochs, batch size 32, and learning rate 0.01; model updates are aggregated as θ^{t+1} = ∑ (n_k / N) θ_k^t. Differential privacy (σ=1.0) is applied via gradient clipping and Gaussian noise during upload.” | We agree that the previous version contained excessive tutorial-style explanations and redundant equations. In the revised manuscript, we have condensed the Federated Learning Implementation section into a single focused paragraph, removed Algorithm 4 and Equations (14–18), and retained only Algorithm 3 for clarity. The revised text now explicitly specifies the federated protocol and hyperparameters, including the number of clients, local epochs, batch size, learning rate, aggregation rule (FedAvg), and differential privacy settings. This revision improves conciseness, technical precision, and alignment with publication standards. |
| 7 | Results need to be improved and more results are required to strengthen the claims.  Currently, It lacks critical context—no baseline names in Figure, no ablation studies, no statistical significance, and no error metrics (sensitivity, specificity, AUC). Add a compact comparison table with specific baselines (e.g., “ResNet-50 centralized: 94.2%, DenseNet-121 FedAvg: 95.1%”) and 95% CI or p-values; include per-dataset breakdown and key clinical metrics (e.g., “Sensitivity: 96.8%, Specificity: 96.5% on Deep-III”). | The required suggestion has been incorporated to strengthen the results section. The results section now include the comparative analysis with based line and a new subsection of ablation study. |
| 8 | The manuscript claims privacy preservation via federated learning but does not provide empirical privacy evaluations (e.g., differential privacy noise impact, data leakage checks).  Include standard deviation of accuracy across datasets and one ablation (e.g., “w/ vs. w/o federated training”, “w/ vs. w/o augmentation”). Replace vague praise (“strong generalization”) with one sentence: “Deep-Fed maintains <0.5% accuracy variance across Deep-I/II/III, outperforming centralized training by 2.1% on average under non-IID conditions.”   Include small, annotated image panel (e.g., Fig. X: “Success/Failure Cases”) to visually demonstrate clinical relevance and model failure modes.  Specially, Improve Figure quality and type. Replace large, outdated bar charts with compact, high-resolution visualizations (e.g., grouped bar plots with error bars or heatmaps) using a clean, modern style (e.g., seaborn/matplotlib with minimal clutter, consistent fonts, and 300+ DPI).  Add only once algorithm as Proposed Model rather thane every step contains one Algorithms with no information.   Write like a paper, not a thesis: Cut the tutorials, equations, and algorithms—no one needs a CNN 101 or FedAvg pseudocode in 2025. Every sentence must advance your contribution, not teach basics. A paper should feel tight and surgical, not a 50-page chapter. A strong idea can die from poor communication: You have good results and a relevant problem, but FractureNet is a ghost, FL is vanilla, and half the text is filler. Define your novelty in one clear sentence, back it with one table and one figure, and let the results speak—don’t bury them in pedagogy. | We thank the reviewer for this valuable suggestion. We acknowledge that visual case analysis can be beneficial in certain diagnostic studies. However, the primary focus of this work is an applied, system-level evaluation of federated learning for accurate fracture classification, rather than visual interpretability or qualitative case analysis. The clinical relevance of the proposed framework is therefore demonstrated through comprehensive quantitative evaluation, including comparisons with established baselines, ablation studies, and clinically meaningful performance metrics across multiple datasets.  Given the scope of the study and space considerations, we believe that the reported quantitative results are sufficient to support the claims of accurate fracture classification within a privacy-preserving federated setting. Nevertheless, we acknowledge the reviewer’s suggestion and consider qualitative visual analysis as a valuable direction for future work.  We thank the reviewer for this suggestion. The proposed framework is implemented in a modular manner, where each component addresses a distinct stage of the pipeline and the output of one module serves as the input to the subsequent module. To maintain clarity and reproducibility, the original manuscript presented algorithmic steps for each module separately.  That said, we agree that the presentation can be streamlined. In the revised manuscript, we have retained a single consolidated algorithm describing the overall proposed model, while removing or merging auxiliary algorithm blocks that do not add new technical information. This revision improves readability while preserving the modular design of the proposed framework. |
