# Supplementary material for: Deep-Fed: A comprehensive solution for precise bone fracture identification in athletes
Source: PLoS One. 2026 Mar 12;21(3):e0343980. doi: 10.1371/journal.pone.0343980 (PMC12981491; doi:10.1371/journal.pone.0343980)
Supplement: Attachment — Submitted filename: Response to Reviewers 3.0.docx [file pone.0343980.s005.docx]

**Response to Editor’s Comments**

**Title: Deep-Fed: A Comprehensive Solution for Precise Bone Fracture Identification in Athletes**

We are very thankful to the editor for his decision and reviewers for their valuable suggestions. We have revised the whole manuscript, incorporated all the comments, and highlighted the changes in the revised draft. We hope that the revised manuscript is now according to the required standard. The response to the reviews is summarized as below:

| **S. No.** | **Reviewers Comments** | **Author Reply:** |
| --- | --- | --- |
| **Reviewer 1** | | |
| 1 | All comments have been addressed | We sincerely thank the reviewer for their valuable feedback. |
| **Reviewer 4** | | |
| 1 | This manuscript is interesting, but the following issues should be addressed:  A formal inferential statistical analysis should be performed to test whether the diagnostic performance of DeepFed was statistically significantly better than the baseline models.  Also in the Abstract accuracy rates should be reported for DeepFed I-II-III and each baseline model along with some variability index (e.g., 95% CI) and the p-values of each statistical comparison. | We sincerely thank the reviewer for their valuable feedback.  A formal inferential statistical analysis has now been performed to compare Deep-Fed with all baseline models. The results, presented in Table 4, show that Deep-Fed significantly outperforms the baselines (p < 0.05), confirming the robustness and superiority of the proposed approach.  The Abstract has been updated to include the accuracy rates for Deep-Fed I–III and all baseline models, along with variability indices (± standard deviation) and corresponding p-values for statistical comparisons, as suggested. |
| 2 | Overall, it it strongly advisable that the manuscript follow a standard format for a scientific journal (i.e., Introduction, Materials and Methods, Results and Conclusions). The Introduction should be shortened by at least 30% by leaving out unnecessary, well-known preliminary information (especially e.g. at lines 26-48), and one sentence should be added to mention the key role of AI tools in fostering the development of imaging biobanks for enhanced precision medicine (see e.g. doi 10.1007/s00330-021-08431-6).  Furthermore, the key concepts of the Literature Review section should be condensed in the revised Discussion section, which should be focused on discussing the key experimental findings of the authors' own study in view of the existing literature, highlighting any relevant comparisons and differences. | The manuscript has been revised to follow the standard scientific journal format, including Introduction, Materials and Methods, Results, and Conclusions.  The Introduction has been shortened by approximately 30% by removing well-known preliminary information regarding the skeletal system. Additionally, we have added a sentence highlighting the key role of artificial intelligence tools in enabling the development of imaging biobanks to support precision medicine and enhance fracture detection (doi: 10.1007/s00330-021-08431-6).  The Discussion section has been revised to condense key concepts from the Literature Review and to emphasize the experimental findings of the proposed Deep-Fed framework in relation to existing studies, highlighting key comparisons, differences, and improvements. |
